# Integrated genome-wide association and transcriptomic analysis to identify receptor kinase genes to stripe rust resistance in wheat germplasm from southwestern China

**DOI:** 10.1186/s12870-024-05020-9

**Published:** 2024-04-24

**Authors:** Liang Qiao, Jianfei Luo, Huiyutang Wang, Yixi Kong, Tingting Du, Peng Qin, Baoju Yang

**Affiliations:** College of Agronomy and Biotechnology, Yunnan Agricultural University, Kunming, 650201 China

**Keywords:** Wheat, Stripe rust, GWAS, Transcriptome, Receptor kinase, Resistance

## Abstract

Stripe rust of wheat, caused by *Puccinia striiformis* f. sp. *tritici* (*Pst*), is one of the most important diseases of wheat worldwide. Identification of new and elite *Pst*-resistance loci or genes has the potential to enhance overall resistance to this pathogen. Here, we conducted an integrated genome-wide association study (GWAS) and transcriptomic analysis to screen for loci associated with resistance to stripe rust in 335 accessions from Yunnan, including 311 landraces and 24 cultivars. Based on the environmental phenotype, we identified 113 protein kinases significantly associated with *Pst* resistance using mixed linear model (MLM) and generalized linear model (GLM) models. Transcriptomic analysis revealed that 52 of 113 protein kinases identified by GWAS were up and down regulated in response to *Pst* infection. Among these genes, a total of 15 receptor kinase genes were identified associated with *Pst* resistance. 11 candidate genes were newly discovered in Yunnan wheat germplasm. Our results revealed that resistance alleles to stripe rust were accumulated in Yunnan wheat germplasm, implying direct or indirect selection for improving stripe rust resistance in elite wheat breeding programs.

**Supplementary Information:**

The online version contains supplementary material available at 10.1186/s12870-024-05020-9.

## Introduction

Wheat (*Triticum aestivum* L.) is one of the three major crops worldwide, providing 20% of the protein and energy required for the global population, making it one of the most important food security crops. The wheat stripe rust caused by *Puccinia striiformis* f. sp. *tritici* (*Pst*) is a devastating fungal disease that threatens wheat production, causing over 40% of wheat yield losses in pandemic years [1, 2]. Among all the available methods of stripe rust management, breeding and growing wheat resistant varieties is the most effective, environmentally-safe and economical way to control the disease.

There are three types of resistance to stripe rust, all-stage resistance (ASR), adult-plant resistance (APR) and high-temperature adult-plant (HTAP) resistance [3]. ASR is race-specific and typically based on a single major gene, which is generally considered to be short-lived due to the emergence of new virulent and more aggressive *Pst* pathotypes. APR is often race non-specific, and usually quantitatively inherited, thus more likely to be durable [4].

Up to now, 86 officially designated stripe rust resistance genes, 71 temporarily designated genes and 363 quantitative trait loci (QTLs) with different names have been reported in wheat. The 86 officially designated stripe rust resistance genes include 22 APR genes and 6 HTAP resistance genes [5, 6]. Nine *Yr* genes have been cloned. Six of these cloned genes were ASR, including *Yr5*/*Yr7/YrSP* [7], *Yr10* [8], *Yr15* [9], *Yr27* [10], *Yr28* (*YrAS2388*) [11] NLR proteins, except *Yr15* which encodes a tandem kinase-pseudokinase. Three cloned APR genes, including *Yr18* [12], *Yr36* [13] and *Yr46* [14], encoding an ATP-binding cassette transporter, a wheat kinase start 1 (WKS1) protein, and a hexose transporter, respectively. The large number of disease resistance genes mentioned above have not been cloned yet, resulting in limited understanding of resistance mechanisms and interactions with the *Pst*.

Most cloned resistance (R) genes in many species encode nucleotide-binding, leucine-rich repeat (NLR) intracellular immune receptors (55%; e.g. *L6* and *Pps2*) or cell-surface receptors (17%; e.g. *Cf-9* and *Xa21*) [15]. Other R genes encode receptor-like cytoplasmic kinases, ubiquitin proteins and transcription factors [16]. However, in wheat and barley, kinase fusion proteins are emerging as key players [17–19].

To identify more candidate R genes to stripe rust, the present study employed 335 wheat accessions and transcriptomic data associated with *Pst* inoculation to detect *Pst*-related candidate genes by integrating genome-wide association study (GWAS) and transcriptomic datasets. The integrated results presented in this study provide resources for optimizing breeding strategies of wheat resistance to *Pst*.

## Materials and methods

### Plant materials and phenotypic data collection

A total of 335 wheat accessions (Hexaploid, Semi winter wheat) from Yunnan, China, including 311 (93%) landraces and 24 (7%) cultivars (Supplementary Table 1), were sown in the experimental field of Yunnan Agricultural University (25°13’N, 102°75’E) and at Xundian County, Kunming (25°20’N, 102°41’E) during cropping seasons, 2018–2019, 2019–2020, 2020–2021, and 2021–2022. Each entry was planted in 1 m row spacing and 25 cm inter-row spacing, with three replications per environment in a randomized block design. The seeds are sown in mid-to-late October each year. Mingxian 169 was planted every 20 rows as the susceptible control and surrounded by a nursery to increase stripe rust pressure. Identification of stripe rust resistance in wheat under natural disease conditions. When the occurrence of stripe rust entered the peak period and wheat heading (around mid-to-late March to early April), the infection types (ITs) of all accessions to stripe rust were recorded on adult plant leaves. Infection types (ITs) were scored using an ordinal scale of disease severity that has been previously developed to characterize the phenotypes of wheat plants following infection of wheat stripe rust pathogens; the ordinal scores range from 0 to 9, where 0 indicates resistance to infection, 1–3 indicate high resistance to moderate resistance, 4–6 indicate intermediate resistance, and 7–9 indicate moderate to high susceptibility [3, 20, 21].

### Genotyping

Seedling leaf samples of all accessions were collected for genomic DNA isolation by the cetyltrimethylammonium bromide method. The genomic DNA was digested with restriction endonucleases MseI and NlaIII, and then barcodes were added to each sample and amplified, then the barcoded samples were pooled and the desired fragments were selected for genotyping by sequencing (GBS) library construction. The Illumina HiSeq sequencing platform was used to conduct 150-bp paired-end sequencing. All reads were processed for quality control and filtered using Seqtk (https://github.com/lh3/seqtk) software. BWA software (v0.7.17) was used to map the filtered sequencing data to the Chinese Spring genome (*Triticum_aestivum*.IWGSC.dna.toplevel.fa; V2). GATK (v4.1.4.0) software was used to identify genome-wide variants, and the “KNN” imputation algorithm in TASSEL (v5.2.60) software was used to impute missing variants in the original dataset (geno < 0.9). The raw data analysis yielded 3,161,158 SNP loci, which were screened for variation using Plink (v1.90 b6.26) software with the parameters “maf > 0.05; geno < 0.4” (minor allele frequency > 0.05, missing genotype data < 0.4), and finally yielded 226,206 high-quality single nucleotide polymorphism (SNP) markers (genome A: 89,457, genome B: 125,531, genome D: 9,190, positional information unknown: 2,028) were obtained for subsequent GWAS.

### GWAS

GWAS was conducted to determine associations between SNP markers and ITs. The software Tassel was used for the kinship matrix analysis. Tassel (v3.0.70) [22] software was used to convert the VCF format files into HMP format for association analysis. Based on the phenotypic infection types to *Pst* and GBS genotypic datasheets of 335 wheat accessions that showed on our reported research [23], a GWAS analysis was performed to ascertain the candidate *Pst* resistance genes by utilizing the mixed linear model (MLM) (K + Q) and generalized linear model (GLM) models in TASSEL software [24, 25]. The MLM and GLM both yielded significant loci with the threshold -log_10_ (*P*) > 4.0 [26], and the CMplot package (https://github.com/YinLiLin/CMplot) was used to build Manhattan and QQ plots.

### RNA-sequencing and transcriptome analysis

According to the study we reported, wheat materials Y0337 (Baikemai-11, Hexaploid, Semi winter wheat) and Y0402 (Batangxiaomai, Hexaploid, Semi winter wheat) were selected for *Pst* inoculation experiments. When wheat plants enter the second leaf stage in the artificial culture chamber, *Pst* strain CYR32 was mixed with talcum powder and inoculated on plant leaves at a ratio of 1:20. Three replicates of Y0337 (average IT of 2) and Y0402 (average IT of 9) leaf samples were harvested at 0, 24, 48, 72, 120 and 168 h post *Pst* CYR32 inoculation for RNA-sequencing. The RNAprep Pure Plant kit DP411 (Tiangen Biotech, China) was used to isolate total mRNA. 1 µg of RNA from each sample was used to construct the complementary DNA (cDNA) library, and the insert fragments of the library were detected using a Qubit 3.0 fluorescence quantifier and Qsep400 high-throughput analysis system. The 150-bp paired-end sequencing was conducted using the Illumina NovaSeq 6000 sequencing platform. The high-quality clean reads were mapped to Chinese Spring RefSeq v2.0 using software HISAT2 [27].

For RNA-seq results, we use StringTie Normalized using FPKM (Fragments Per Kilobase of transcript per Million fragments mapped) by the maximum flow algorithm as a measure of transcript or gene expression level [28]. Differentially expressed gene analysis was performed using DESeq2 [29] software. Differentially expressed genes (DEGs) were obtained by comparing between samples at different time periods after inoculation with stripe rust spores, and genes that also met padj ≤ 0.01 and |log_2_(FoldChange)|≥ 1 were considered as DEGs. Gene Ontology (GO) and Kyoto Encyclopedia of Genes and Genomes (KEGG) enrichment analyses were performed on the differential genes. GO and KEGG analyses were conducted using the R package ClusterProfiler (version 4.0.0) [30].

### RT-qPCR

The samples used in RT-qPCR were the same as those used for RNA-seq. An Aidlab Reverse Transcription Kit (TUREscript 1st Strand cDNA Synthesis Kit, Beijing, China) was used to synthesize cDNA. An Analytik Jena qTOWER 2.2 fluorescent quantitative PCR instrument (Jena, Germany) with 2 × SYBR® Green Supermix (Biomarker Technologies, Beijing, China) was used to conduct RT-qPCR reactions. The primer sequences are shown in Supplementary Table 2. The 2^−∆∆Ct^ method was used to calculate the expression levels of genes [31]. The GAPDH gene was used as the internal control [32]. The correlation analysis of RT-qPCR and RNA-seq and the drawing of scatter plots were carried out using an Excel table.

### Integrated analysis of GWAS and transcriptomics data

The results of GWAS and RNA-seq analysis were combined to further screen candidate genes. Heatmapping of candidate gene expression was performed using the R package Pheatmap (version 4.0.0). The trend of FPKM expression of candidate genes at each time point was plotted and analyzed by ANOVA with a Tukey post hoc test using Graphpad Prism (version 9.0) software. Physical maps of previous research QTLs and candidate genes from this study were utilized with the R package LinkageMapView (version 4.0.0).

## Results

### GWAS analysis of *Pst* resistance

GLM and MLM models were used for GWAS analysis of *Pst* phenotypic infection types and GBS genotype data sheets. Both the MLM and GLM were used to identify significant loci (-log_10_ (P) > 4.0), and the threshold for statistical significance was *P* = 1e-4.0. A GWAS analysis of wheat stripe rust resistance showed that 3475 SNP markers (Supplementary Table 3) exceeding the threshold were detected on 21 chromosomes. The significant SNP marker associated genes were selected based on GO and KEGG enrichment analysis, and a total of 113 protein kinase genes were identified (Supplementary Table 4). Among these candidate genes, 7 genes were found in at least two environments (Supplementary Table 4).

### Transcriptomic analyses of *Pst*-induced changes in gene expression

According to our reported study [23], more than 80% of the DEGs obtained from the transcriptomes of Y0337 and Y0402 samples were concentrated at the 24 hpi and 48 hpi time points, and the disease-resistant variety Y0337 had more than twice as many DEGs as the susceptible variety Y0402 at the 24 hpi and 48 hpi time points. The wheat resistance response was the strongest at 24 h–48 h after inoculation with stripe rust, and more genes were involved in the regulation of the response in disease-resistant varieties after inoculation with stripe rust.

**Fig. 1:**
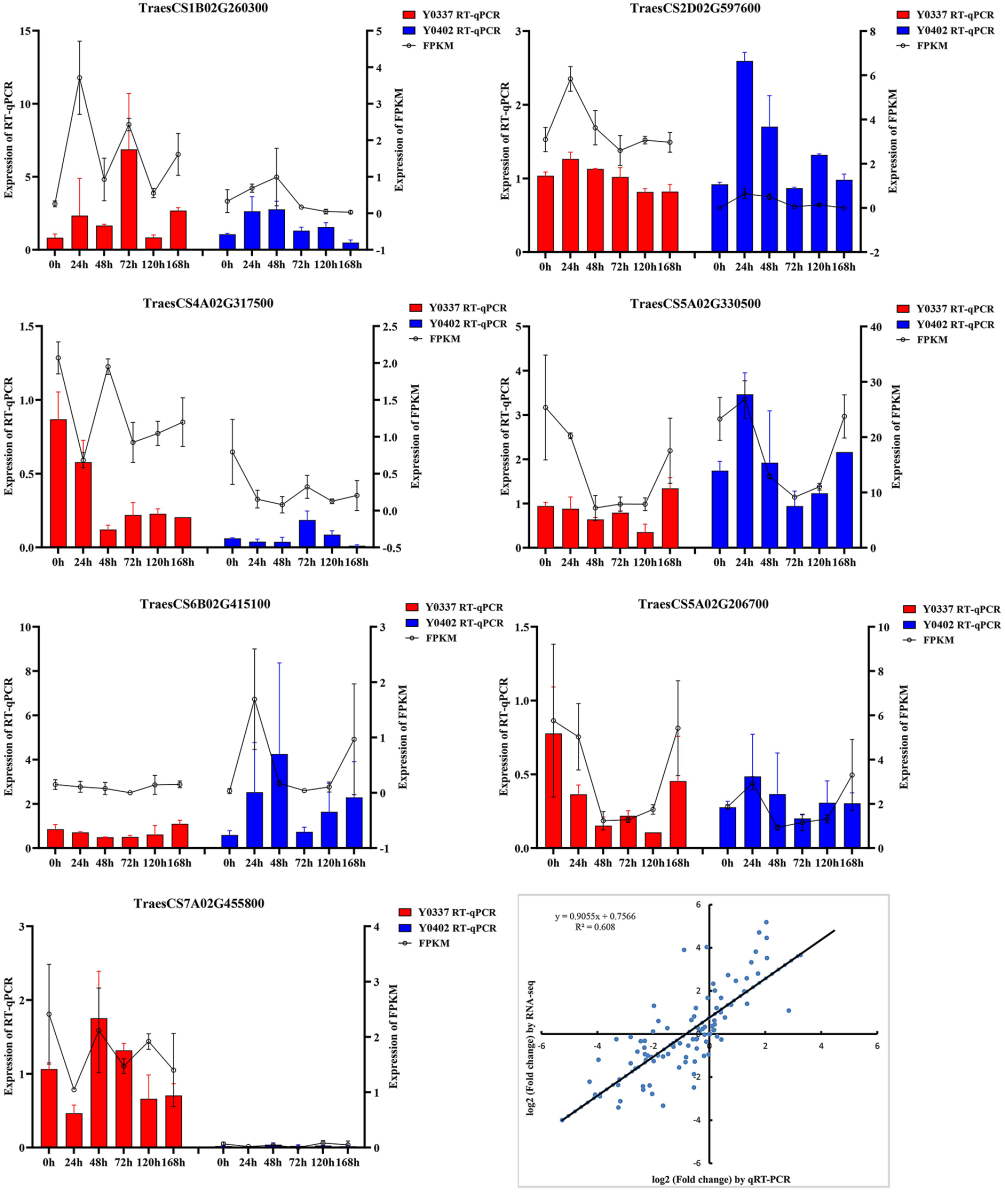
Analysis of expression profiles of 7 kinase genes post *Pst *inoculation . The bar graph with standard error is shown as the relative expression levels corresponding to three independent biological replicates measured by 2^-△△CT^ RT-qPCR. The solid line indicates the expression level of the sample FPKM

We conducted RT-qPCR analysis of seven randomly selected DEGs from both the Y0337 and Y0402 samples to validate the RNA-seq data. Correlation analysis showed a high correlation between the RNA-seq data and RT-qPCR data (R^2^ of 0.608), suggesting the RNA-seq data were reliable (Fig. 1).

**Fig. 2:**
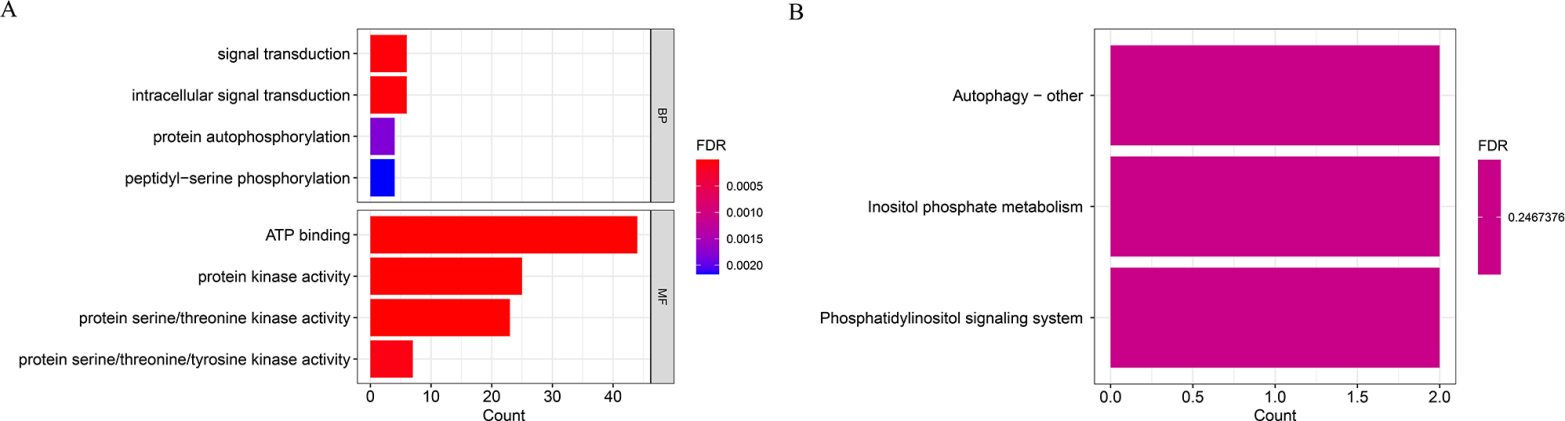
Enrichment analysis of 52 protein kinase genes: A. GO enrichment analysis, B. KEGG enrichment analysis. BP: Biological Process. MP: Molecular Function

Transcriptome sequencing revealed that 52 of 113 protein kinases identified by GWAS were up- and downregulated in response to *Pst* infection (Supplementary Table 5). GO analysis showed that the 52 DEGs were significantly enriched in ATP binding, protein kinase activity, protein serine/threonine kinase activity and protein serine/threonine/tyrosine kinase activity (Fig. 2A). KEGG analysis further showed that these DEGs were enriched in multiple metabolic pathways, including signal transduction, intracellular signal transduction, protein autophosphorylation and peptidyl-serine phosphorylation pathways (Fig. 2B).

### Integrated analyses of GWAS and transcriptomic data

**Fig. 3:**
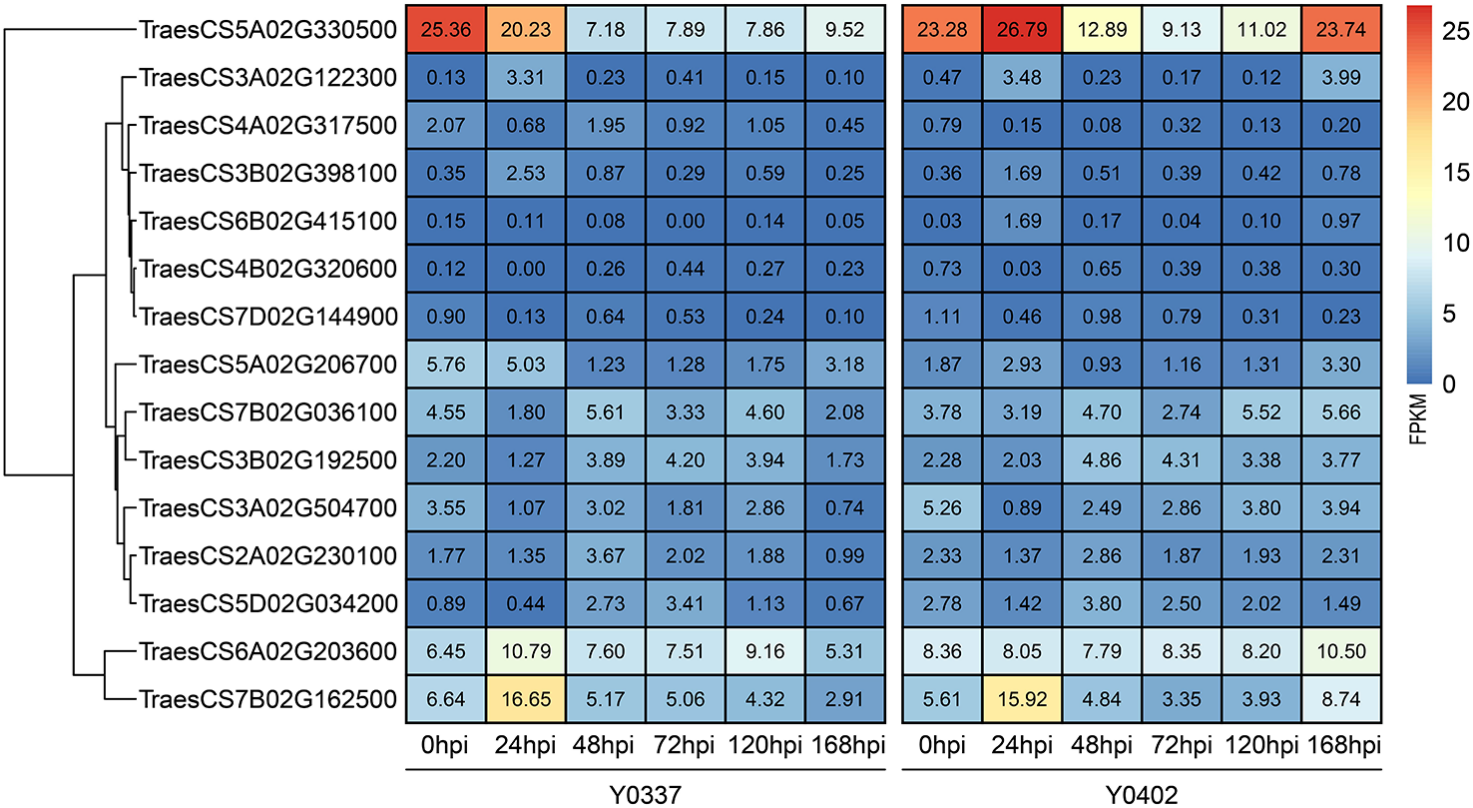
Heatmap analysis of expression of 15 candidate resistance genes in response to infection of wheat by *Pst*. Colors indicate the FPKM expression levels of the genes in the transcripts, and blue to red indicate low to high FPKM expression levels

**Fig. 4:**
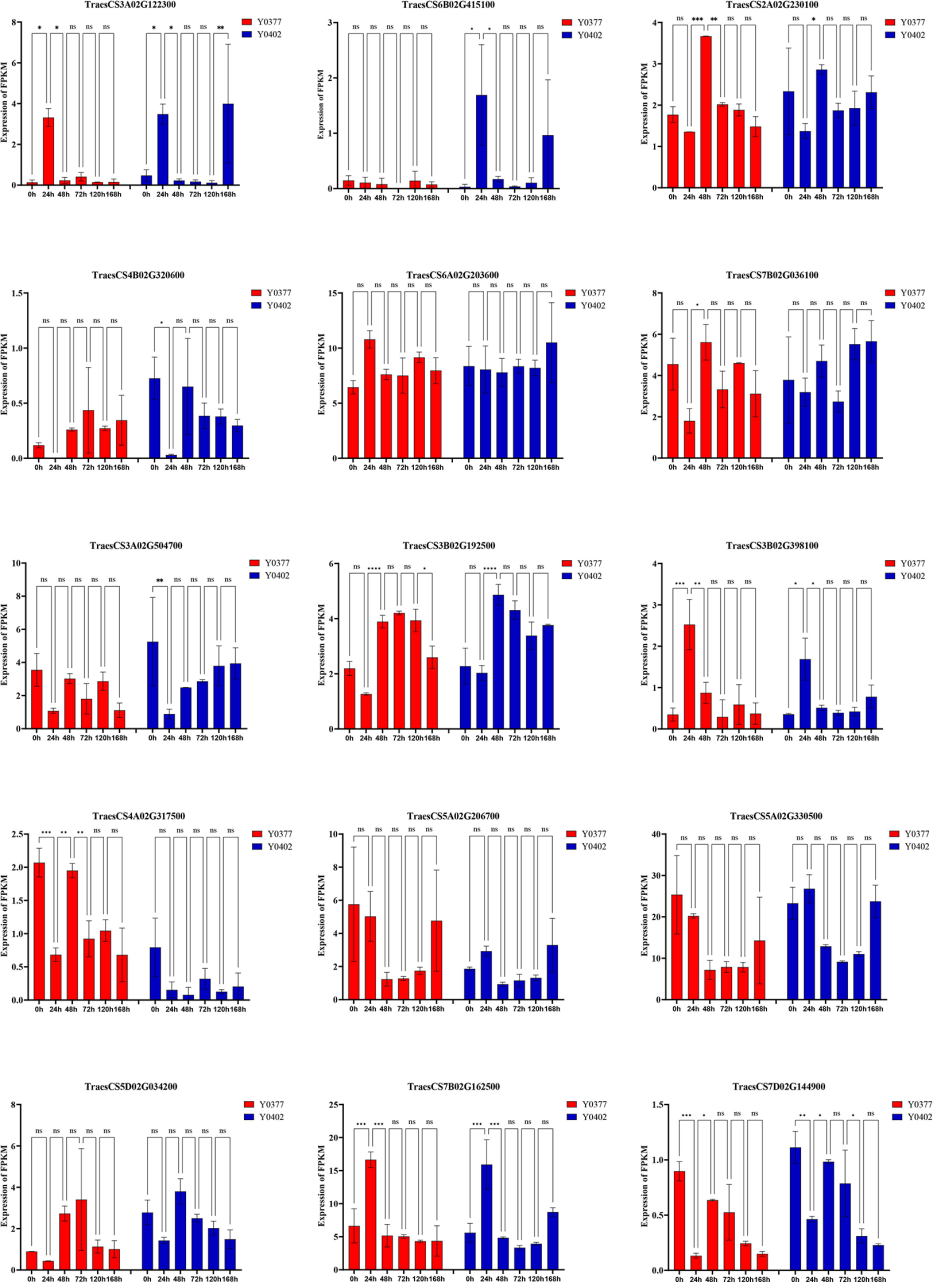
Expression patterns of fifteen candidate genes for wheat stripe rust resistance following inoculation of Y0377 and Y0402 with *Pst*. The red bar shows the FPKM expression of sample Y0377, and the blue bar shows the FPKM expression of sample Y0402

To comprehensively investigate candidate genes to *Pst* resistance, the findings obtained from GWAS and RNA-seq analyses were combined. The Integrated analyses detected 15 receptor kinase genes as the *Pst* resistance genes, including 2 cell wall-associated receptor kinase genes, 4 LRR-receptor kinase genes and 9 receptor-like kinase genes (Table 1). The expression heatmap of these genes is shown in Fig. 3, in which the FPKM expression of the *TraesCS5A02G330500*, *TraesCS6A02G203600*, and *TraesCS7B02G162500* genes were relatively high. ANOVA analysis of the expression levels between time points showed that the most significant differences in the expression of the transcript genes were observed at 24 h after inoculation with stripe rust (Fig. 4), and that the strongest regulatory responses for resistance genes occurred in wheat at about 24 h after inoculation with stripe rust. *TraesCS5A02G330500*, *TraesCS4A02G317500*, *TraesCS6B02G415100*, *TraesCS4B02G320600*, *TraesCS7D02G144900*, *TraesCS5A02G206700*, *TraesCS7B02G036100*, *TraesCS3B02G192500*, *TraesCS3A02G504700*, *TraesCS2A02G230100*, and *TraesCS5D02G034200* genes were down-regulated in terms of expression at 24 h after inoculation with stripe rust. *TraesCS3A02G122300*, *TraesCS3B02G398100*, *TraesCS6A02G203600*, and *TraesCS7B02G162500* genes were up-regulated in terms of 24hpi expression profiles.

**Fig. 5:**
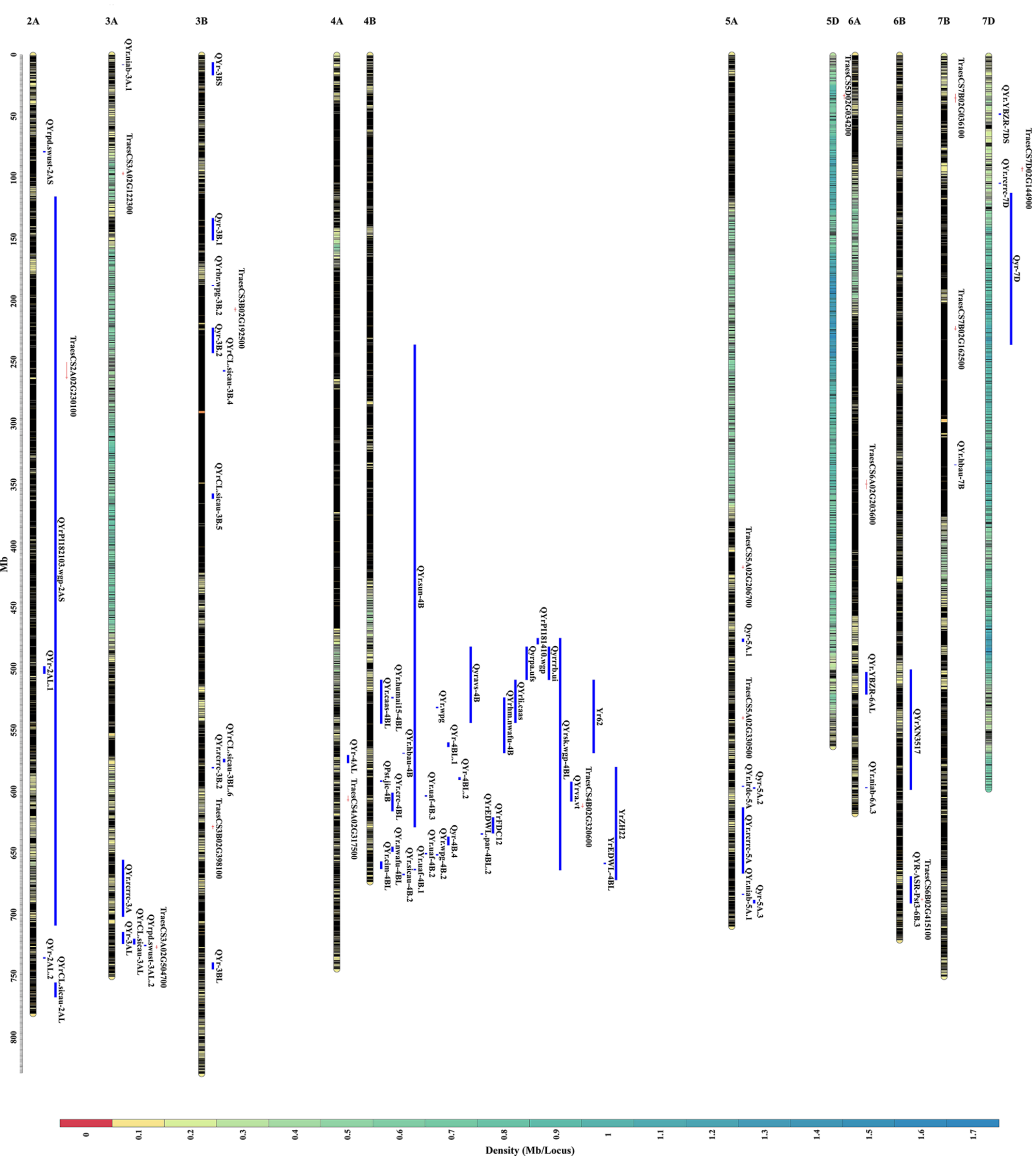
Genetic linkage map of the *Yr* genes /QTLs in wheat. Vertical coordinates indicate the physical location of the chromosome; black annotations on the chromosome indicate the SNP locus; and the color indicates the density of the SNP locus (Mb/Locus); red to blue indicates the density from low to high. In the figure, the extra-chromosomal blue standards are the physical locations of QTLs studied by previous researchers, and the red markers are the physical locations of candidate genes in this study

The 15 candidate genes were distributed on chromosomes 2A, 3A, 3B, 4A, 4B, 5A, 5D, 6A, 6B, 7B and 7D. Checking the physical maps of previous studies of QTL in related literature and candidate genes in this study (Fig. 5), four genes, *TraesCS2A02G230100*, *TraesCS3A02G504700*, *TraesCS4B02G320600*, and *TraesCS6B02G415100*, overlapped with the locations of the QTL in previous studies. The gene *TraesCS2A02G230100* overlapped with the position of *QYrPI182103.wgp-2AS*. The gene *TraesCS3A02G504700* is close to the position of *QYrpd.swust-3AL.2*. The gene *TraesCS4B02G320600* overlapped with the positions of *QYr.sun-4B*, *QYrsk.wgp-4BL*, *YrZH22*, and *QYr.crc-4BL*. The gene *TraesCS6B02G415100* overlapped with the position of *QYR-ASR-Pst3-6B.3*. No previous QTL-related studies were reviewed near the 11 candidate genes such as *TraesCS3A02G122300*, which may be possible new resistance genes.

**Table 1:** Identification of 15 candidate resistance genes to *Pst* infection

ID	GWAS	Chr	Gene.start.bp.	Gene.end.bp.	Gene annotation
*TraesCS2A02G230100*	2019XD-GLM	2AS	262,573,269	262,577,316	LRR receptor-like serine/threonine-protein kinase GSO1
*TraesCS3A02G122300*	2022XD-GLM	3AS	96,682,449	96,685,033	wall-associated receptor kinase-like 20
*TraesCS3A02G504700*	2022YN-MLM	3AL	726,740,992	726,743,961	receptor-like protein kinase FERONIA
*TraesCS3B02G192500*	2020YN-GLM	3BS	207,120,527	207,124,401	receptor-like protein kinase HSL1
*TraesCS3B02G398100*	2020YN-GLM	3BL	628,469,977	628,475,070	calmodulin-binding receptor-like cytoplasmic kinase 2
*TraesCS4A02G317500*	2020YN-GLM	4AL	606,761,092	606,779,802	cysteine-rich receptor-like protein kinase 15
*TraesCS4B02G320600*	2020YN-GLM	4BL	612,024,567	612,027,930	LRR receptor-like serine/threonine-protein kinase
*TraesCS5A02G206700*	2019XD-GLM	5AL	417,252,341	417,257,021	receptor-like protein kinase
*TraesCS5A02G330500*	2019YN-GLM	5AL	540,050,948	540,055,082	receptor-like protein kinase FERONIA
*TraesCS5D02G034200*	2020YN-GLM	5DS	33,022,143	33,026,691	inactive receptor kinase
*TraesCS6A02G203600*	2019YN-GLM	6AL	349,509,306	349,518,822	LRR receptor kinase SERK2-like
*TraesCS6B02G415100*	2019XD-GLM	6BL	687,954,847	687,957,416	wall-associated receptor kinase 5-like
*TraesCS7B02G036100*	2020YN-GLM	7BS	35,081,317	35,085,883	LRR receptor kinase SERK2-like
*TraesCS7B02G162500*	2020YN-GLM	7BS	223,172,017	223,178,821	chitin elicitor receptor kinase 1-like
*TraesCS7D02G144900*	2019XD-GLM	7DS	92,568,812	92,575,801	receptor kinase-like protein Xa21

## Discussion

### Wheat landraces from Yunnan for *Pst* resistance

Yunnan Province is located in the southwestern wheat region of China, which is one of the major “over-summering areas” for wheat rust. Moreover, Yunnan is also a crucial area for race variation owing to the complex topography and diverse climate types that facilitate the virulence variation of *Pst* [33]. Previous researchers have focused on stripe rust resistance of local wheat varieties in Yunnan, and found a large number of germplasms with strong resistance to stripe rust; the results of molecular identification indicated that there might be some unknown new stripe rust resistance genes or combinations in Yunnan wheat local varieties, which could provide new resistance sources for wheat breeding with persistent resistance to stripe rust. The resistance of 63 Yunnan wheat landraces to stripe rust was identified by Li et al. [34] and the results showed that 35 showed resistance and 28 showed slow rust, which can be used as excellent resistant genetic materials for wheat quality improvement and breeding. Chen et al. [35] identified the resistance of 260 Yunnan wheat landraces to stripe rust at the adult plant stage and the genotypes of three known resistance genes, *Yr5*, *Yr10*, and *Yr15* were determined, the results suggested that there are abundant stripe rust resistant materials in Yunnan’s local wheat resources. A total of 131 immune, near-immune, and high-resistant materials were also screened, and none of the tested materials carried the known resistance genes, *Yr5* and *Yr15*. Xi et al. [36] analyzed the resistance of 243 Yunnan local wheat varieties to stripe rust at seedling and planting stages, and screened 174 materials with stable resistance at planting stage, the 16 known resistance genes including *Yr5*, *Yr10*, *Yr15*, *Yr18*, *Yr26*, *Yr28*, *Yr29*, *Yr30*, *Yr36*, *Yr39*, *Yr41*, *Yr48*, *Yr65*, *Yr67*, *Yr80* and *Yr81*, were not identified in 58 resistance materials. The resistance of 78 Yunnan iron-shelled wheat to stripe rust were characterized by Li et al. [37], all the materials showed resistance at the adult stage. No known resistance genes such as *Yr5, Yr9, Yr10, Yr15, Yr17, Yr18, Yr24/Yr26, Yr30, Yr41, Yr48, Yr65*, and *Yr67* were identified. These results further suggested that Yunnan-specific wheat germplasm may carry other known or new stripe rust resistance genes, and is an important source material for cultivating wheat varieties with durable resistance to stripe rust.

### Comparing the 15 candidate genes associated with *Pst* with the previous *Pst* QTL

In this study, a total of 15 candidate genes associated with *Pst* were identified by integrating GWAS and transcriptome datasets of 335 wheat cultivars inoculated with *Pst*. The 15 candidate genes were distributed on chromosomes 2A, 3A, 3B, 4A, 4B, 5A, 5D, 6A, 6B, 7B, and 7D. By comparing the QTL positions reported in previous studies (Fig. 5, Supplementary Table 6), the candidate gene *TraesCS2A02G230100* (located at chromosome 2A at 262,573,269 bp) overlapped with the position of *QYrPI182103.wgp-2AS* [38], which is a QTL locus from Pakistani accession PI182103 , and the locus can be detected at the seedling stage of wheat. The candidate gene *TraesCS3A02G504700* (located at 726,740,992 bp on chromosome 3 A) is close to the position of *QYrpd.swust-3AL.2* [39], which is located between markers IWA95 and IWB13994, localizes to CS chromosome 3AL at 724,201,099 bp-725,738,951 bp, and confers APR resistance. The candidate gene *TraesCS4B02G320600* (located at 612,024,567 bp on chromosome 4B) overlapped with the positions of *QYr.sun-4B*, *QYrsk.wgp-4BL*, *YrZH22*, and *QYr.crc-4BL* [40–43] , in which *QYr.sun-4B* and *QYr.crc-4BL* were micro efficiency loci inherited from synthetic hexaploid CPI133872  and cultivar Toropi, respectively, and *QYrsk.wgp-4BL* and *Yr62* might be different alleles on the same locus. The candidate gene *TraesCS6B02G415100* (located at 687,954,847 bp on chromosome 6B) overlapped with the position of *QYR-ASR-Pst3-6B.3* [44], which was identified as the ASR gene locus. The other 11 candidate genes do not overlap with QTL loci reported in previous studies and may be new disease resistance genes.

### *Pst-*induced immune genes in wheat

The vulnerability of resistant wheat varieties to pathogenic variation of stripe rust - pathogen variation can lead to resistant varieties becoming susceptible, which poses a continuous threat to global wheat production and food security. Identifying novel durable resistance resources is important for sustainable control of stripe rust. It is now widely recognized by scholars that resistance genes with LRR structural domains play an important role in regulating plant resistance to pathogens and insects [45]. Many LRR-RLKs have been shown to play key roles in plant immune signaling, where plants recognize various pathogens and activate immune responses through receptor-like kinases (RLKs). Yan et al. [46] have demonstrated that the F-box/LRR protein COI1 is directly involved in defense responses as the jasmonic acid receptor in *Arabidopsis thaliana*. *Traes_4BS_C868349E1*, which encodes the F-box/LRR protein, was indicated to be a key candidate gene for stripe rust resistance in wheat mutant R39, which activates wheat defense responses by regulating hormonal signals such as jasmonic acid and abscisic acid [47]. A previous study showed that TuRLK1 is required for the immune response to stripe rust mediated by the NLR protein YrU1, and may also play an important role in resistance to other pathogens such as powdery mildew, where the expression of the leucine-rich repetitive receptor-like kinase TuRLK1 is upregulated in wheat infected with *Pst* CYR33 [48]. In addition, the leucine repeat receptor-like kinase (LRR-RLK) gene* TaBIR1*, a cell-surface RLK, is suggested to contribute to wheat stripe rust resistance, and may act as a positive regulator of plant immunity in a BAK1-dependent manner [49]. Receptor-like kinase genes have a crucial role in stripe rust resistance in Wheat High-temperature seedling plant (HTSP) resistance, and Wang et al. [50] have shown that TaXa21 is an RLK related to TaWRKY76 and TaWRKY62 and acts as a positive regulator of *Pst* resistance in wheat HTSP resistance. Wang et al. [51] identified a protein kinase CRK gene in wheat *TaCRK10* that plays a positive role in wheat HTSP resistance to *Pst*, and the elevated expression of *TaCRK10* induced by high temperature contributes to wheat HTSP resistance to *Pst*. Among the cloned stripe rust resistance genes, *Yr36* which encodes a kinase and provides stripe rust resistance at the seedling and adult plant stages under relatively high temperatures [13]. In this study, several receptor-like kinase genes significantly associated with stripe rust resistance were detected by integrating GWAS and transcriptome datasets, and these genes can be used as the candidate genes related to *Pst* for further functional studies.

## Conclusions

In this study, based on four years data two-site environmental phenotypes of wheat germplasm from Yunnan, China, we integrated GWAS and transcriptomic analysis to identify 15 receptor kinase genes associated with stripe rust resistance. Compared with the reported *Pst* QTL loci, among the 15 candidate genes, *TraesCS2A02G230100* might have the same position as the *QYrPI182103.wgp-2AS*. The location of *TraesCS3A02G504700* is close to *Qyrpd.swust-3AL.2*, and *TraesCS4B02G320600* might have the same position as *QYr.sun-4B*, *QYrsk.wgp-4B*L, *YrZH22*, and *QYr.crc-4B*. The candidate gene *TraesCS6B02G415100* was located at the position of *QYR-ASR-Pst3-6B.3*, which was identified as the ASR gene QTL locus. The other 11 receptor kinase genes associated with stripe rust resistance were distant from previously identified stripe rust resistance genes or QTL regions, indicating that they may be novel resistance genes. Dissection of genes from the newly observed *Pst* resistance genes can provide new resources of *Pst* resistance genes for wheat breeding.

### Electronic supplementary material

Below is the link to the electronic supplementary material.

Supplementary Material 1

## Data Availability

The original contributions presented in the study are publicly available. The data can be accessed at the following link:https://www.ncbi.nlm.nih.gov/search/all/?term=PRJNA938609.

## Abbreviations

GWAS: Genome-wide association study
MLM: Mixed linear model
GLM: Generalized linear model
ASR: All-stage resistance
APR: Adult-plant resistance
HTAP: High-temperature adult-plant
HTSP: High-temperature seedling plant
QTLs: Quantitative trait loci
WKS1: Wheat kinase start 1
NLR: Leucine-rich repeat
IT: Infection type
DEGs: Differentially expressed genes
GO: Gene Ontology
KEGG: Kyoto Encyclopedia of Genes and Genomes
RLK: Receptor-like kinases
